# Effects of perinatal exposure to nonylphenol on delivery outcomes of pregnant rats and inflammatory hepatic injury in newborn rats

**DOI:** 10.1590/1414-431X20165647

**Published:** 2016-12-08

**Authors:** J. Yu, Y. Luo, X.F. Yang, M.X. Yang, J. Yang, X.S. Yang, J. Zhou, F. Gao, L.T. He, J. Xu

**Affiliations:** 1 School of Public Health, Zunyi Medical University, Zunyi, Guizhou, China; 2 Department of Gastrointestinal Surgery, Affiliated Hospital of Zunyi Medical University, Zunyi, Guizhou, China; 3 Department of Endocrinology, The First Affiliated Hospital of Zunyi Medical University, Zunyi, Guizhou, China

**Keywords:** Nonylphenol, Hepatic injury, Delivery, Newborn rats

## Abstract

The current study aimed to investigate the effects of perinatal exposure to nonylphenol (NP) on delivery outcome of pregnant rats and subsequent inflammatory hepatic injury in newborn rats. The pregnant rats were divided into 2 groups: control group (corn oil) and NP exposure group. Thirty-four pregnant rats were administered NP or corn oil by gavage from the sixth day of pregnancy to 21 days postpartum, with blood samples collected at 12 and 21 days of pregnancy and 60 days after delivery. The NP concentration was measured by HPLC, with chemiluminescence used for detection of estrogen and progesterone levels. Maternal delivery parameters were also observed. Liver and blood of the newborn rats were collected and subjected to automatic biochemical detection of liver function and blood lipid analyzer (immunoturbidimetry), and ultrastructural observation of the hepatic microstructure, with the TNF-α and IL-1β hepatic tissue levels evaluated by immunohistochemistry. Compared with the control group, the pregnant and postpartum serum NP and estradiol levels of the mother rats in the NP group were significantly increased, together with lowered progesterone level, increased number of threatened abortion and dystocia, and fewer newborn rats and lower litter weight. Serum and hepatic NP levels of the newborn rats measured 60 days after birth were significantly higher than those of the control group, as well as lower testosterone levels and increased estradiol levels. When observed under electron microscope, the hepatocyte nuclei of the control group were large and round, with evenly distributed chromatin. The chromatin of hepatocytes in the NP group presented deep staining of the nuclei, significant lipid decrease in the cytoplasm, and the majority of cells bonded with lysate. The results of immunohistochemistry showed that there was almost no TNF-α or IL-1β expression in the hepatocytes of the control group, while the number of TNF-α-, PCNA-, and IL-1β-positive cells in the NP group was increased, with higher integral optical density than the control group. Compared to the control group, the serum levels of alanine aminotransferase, aspartate aminotransferase, triglyceride and low-density lipoprotein in the newborn rats of the NP group were significantly increased. There was no significant difference in the serum level of high-density lipoprotein or cholesterol between the groups. Perinatal exposure to NP can interfere with the *in vivo* estrogen and progesterone levels of pregnant rats, resulting in threatened abortion, dystocia and other adverse delivery outcomes. High liver and serum NP levels of the newborn rats led to alteration of liver tissue structure and function. The NP-induced hepatotoxicity is probably mediated by inflammatory cytokines TNF-α and IL-1α.

## Introduction

Nonylphenol (NP) is a class of environmental endocrine disrupting chemicals (EDCs) interfering with endocrine metabolism by mimicking estrogen and binding with estrogen receptors, which could lead to toxic effects (1). Estrogen and progesterone are two endocrinal hormones closely related to the process of pregnancy and childbirth; alterations of these hormones may lead to adverse outcomes such as abortion, premature birth, stillbirth and postterm pregnancy (2). This study explores two aspects: if the pseudo-estrogen effects of NP interfere with the balance of estrogen and progesterone in the maternal body at the perinatal period and affect delivery; and if perinatal NP exposure can pass through the placenta and be secreted into the breast milk to enter the body of newborn rats. Since liver is the target organ of NP, and is where it is metabolized and accumulated, will it cause inflammatory injury of the liver? If yes, what is the mechanism? These questions have never been addressed in previous reports. Therefore, the current study established a model of perinatal NP exposure and evaluated the changes in hormone levels in the body of mother rats during pregnancy and after delivery, as well as observed the outcomes of delivery. Also, the serum and liver concentrations of NP and the biochemical parameters of liver function and blood lipid of the newborn rats were evaluated, as well as the evaluation of the hepatic TNF-α and IL-1β expression changes, to analyze their correlation with the hepatic NP content and explore the mechanism of NP liver toxicity.

## Material and Methods

### Instruments

HP-1100 HPLC with eclipse plus C8 (5 μm) and 4.6×150 mm (Agilent, USA) was used. Automatic chemical luminescence immunoassay analyzer Centaur XP was purchased from Siemens (Germany). The image analysis system DM 2500 was obtained from Leica (Germany). The automatic biochemical analyzer SYNCHRON, CX 9 PRO was from Beckman Coulter (USA). The pure water system Purelab Ultra Biosci was bought from ELGA (UK). The electronic analytical balance used was a FA2004N model (Shanghai Jingke Balance Instrument Plan, China).

### Reagents

NP for intragastric administration (purity of 98%) was purchased from West Asia, China), HPLC grade NP standard (99.9% purity) from Fluka (Switzerland), corn oil from Luhua (China), estrogen and progesterone detection Kit from Siemens, TNF-α and IL-1β rabbit anti-rat antibody from Beijing Zhongshan Biologicals, China; secondary rabbit anti rat TNF-α and IL-1β polyclonal antibody from Beijing Zhongshan Jinqiao Biologicals; DAB Color Kit from Dako company (Japan), acetonitrile (HPLC grade) from DIKMA Technologies Inc., USA, acetic acid (HPLC grade) from Tianjin Kermel (China), and pure water (YiBao, China).

### Experimental animals

Forty clean grade female adult Sprague Dawley rats and 20 male rats (weight ∼200 g), purchased from the animal center of the Third Military Medical University (animal certification number: SCXK (Chongqing) 2012-0005), were used for mating. Housing conditions were 22°C, and free access to drinking water and food.

### Grouping and NP exposure

After 1 week of housing adaptation, the male and female were put in the same cage at a ratio of 1:2, and the female rats with a large number of sperm observed in vaginal smears under microscope were regarded as pregnant with day 0 gestation. After being stratified based on date of pregnancy, the 34 pregnant rats obtained were randomly divided into two groups: the control group (n=16) and NP group (n=18, intragastric administration of 200 mg·kg^-1^·day^-1^ NP from the 6th day of pregnancy to the 21st day after giving birth). The pregnant rats were sacrificed in two batches, on gestational day 12 and 1 day postpartum, and the serum levels of NP, estrogen and progesterone were measured. Male pups in the litter (8–14/group) were subjected to the measurement of liver function and blood lipid on postnatal day 60.

### Delivery parameters of the pregnant rats

Number and sex ratio of abortions, dystocias and deliveries of the mother rats were recorded, and litter weight and breastfeeding condition of the newborn rats were observed.

### Detection of NP content in serum

For each 0.5 mL of serum or liver supernatant, 4 mL n-hexane and diethyl ether extraction agents (volume ratio of 7:3) was added, vortexed for 30 s, and after resting for 15 min, the supernatant was dried in 50°C water bath and dissolved in 0.5 mL of acetonitrile for analysis. The liquid chromatography conditions used were: fluorescence detector, excitation wavelength of 275 nm, emission wavelength of 312 nm, mobile phase was acetonitrile and acetic acid (v/v, 85:15), with injection volume of 10 µL and flow rate of 1 mL/min.

### Detection of serum levels of estrogen and progesterone

The direct chemiluminescence method was employed in a 2 mL serum sample. Centaur XP Immunoassay System ADVIA (Germany) automatically completed procedures like sampling, incubation, separation, attraction, washing, and initiated the chemiluminescence reaction to measure the hormone content in the samples.

### Evaluation of liver function and blood lipid (Immune turbidimetric method)

A fully automatic biochemical analyzer was used to measure the serum level of AST, ALT, triglyceride, cholesterol, low-density lipoprotein (LDL) and high-density lipoprotein (HDL). The specific steps were conducted according to the manufacturer’s instructions.

### Hepatic ultrastructure electron microscopy

Hepatic tissue of the newborn rats was collected on ice, and then placed on a clean wax plate, with a drop of cooled fixing agent added and quickly cut into 1 mm^3^ pieces. Two percent glutaraldehyde and 1% osmic acid were added for fixation, and the plate was then embedded in epoxy resin 618, subjected to double staining with uranium and lead, and observed under a transmission electronic microscopy (Philips, CM10, 100 KV, Netherland) for the evaluation of ultrastructural changes.

### Expression of TNF-α and IL-1β in liver tissue

*Slice preparation*. 1) The sample was fixed in 4% paraformaldehyde overnight, and subjected to conventional paraffin embedding; 2) xylene gradient dewaxing for 2×10 min; 3) gradient dehydration with anhydrate alcohol, 95% alcohol, and 75% alcohol; 4) 1 min rinse with distilled water; 5) immersed in 3% H_2_O_2_ for 10 min; 6) 0.01 M PBS vibrational flushing for 3×10 min; 7) citrate high-pressure repair for 5 min, keeping warm for 30 min, followed by natural cooling; 8) 0.01 M PBS vibrational flushing for 3×3 min; 9) 30 µL animal serum added and kept at 37°C for 30 min; 10) 30 µL primary anti-TNF-α and IL-1β (1:100 dilution) antibody and kept overnight at 4°C; 11) 0.01 M PBS vibrational flushing for 3×3 min; 12) biotin labeled Goat anti-rabbit IgG added, and incubation at 37°C for 60 min; 13) 0.01 M PBS vibrational flushing for 3×3 min; 14) DAB coloring agent added and incubation for 3–5 min; coloring degree under light microscope observed, after which the coloring was terminated by flushing with tap water; 15) hematoxylin staining for 2 min followed by differentiation using hydrochloric acid and alcohol; 16) flushing with tap water for 10 min; 17) gradient alcohol flushing for 30 s (75–95% alcohol – dehydrate ethanol); 18) mounting with neutral resin adhesive.

### Image analysis

For each slice, 5 fields were randomly selected for analysis (400 × magnification). Image Pro Plus 6.0 software (Media Cybernetics, USA) was used to calculate the number of positive cells and the integral optical density (IOD) value, with higher values suggesting greater staining intensity.

### Statistical analysis

SPSS18.0 was employed for data analysis. The results are reported as mean±SD, with independent samples *t*-test used for comparison between the control and NP group. P<0.05 was considered to be statistically significant.

## Results

### Body weight changes of the pregnant rats

**Figure 1:**
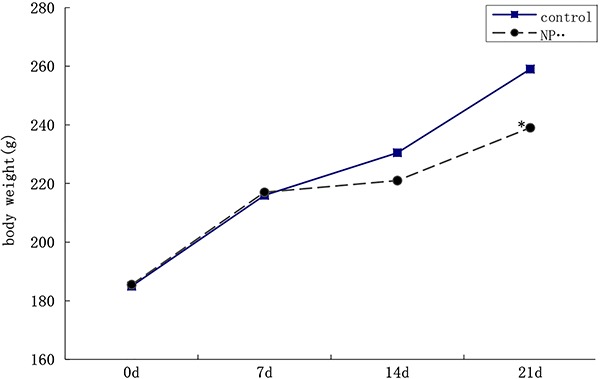
Mean body weight change of pregnant rats administered nonylphenol (NP) or corn oil (control) by gavage from the sixth day (d) of pregnancy. *P<0.05 *vs* control (*t*-test).

As shown in Figure 1, the body weight of the pregnant rats during the first week of pregnancy (before intragastric NP administration) demonstrated a tendency to increase in both groups. During the second week of pregnancy (the first week of intragastric administration), body weight increase was slightly less. From the second week of pregnancy until delivery body weight continued to increase, with rats in the NP group increasing significantly less than the control group.

### Delivery outcome of the pregnant rats

The number of newborn rats and litter weight of the NP group were both lower than those of the control group (P<0.05). Also, the NP group had higher number of threatened abortions and actual dystocia, as well as higher proportion of female newborn rats (Table 1).

**Table:**
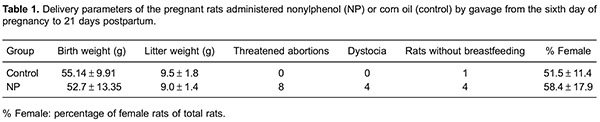

### Serum hormones and NP levels in the mother and newborn rats

**Figure 2:**
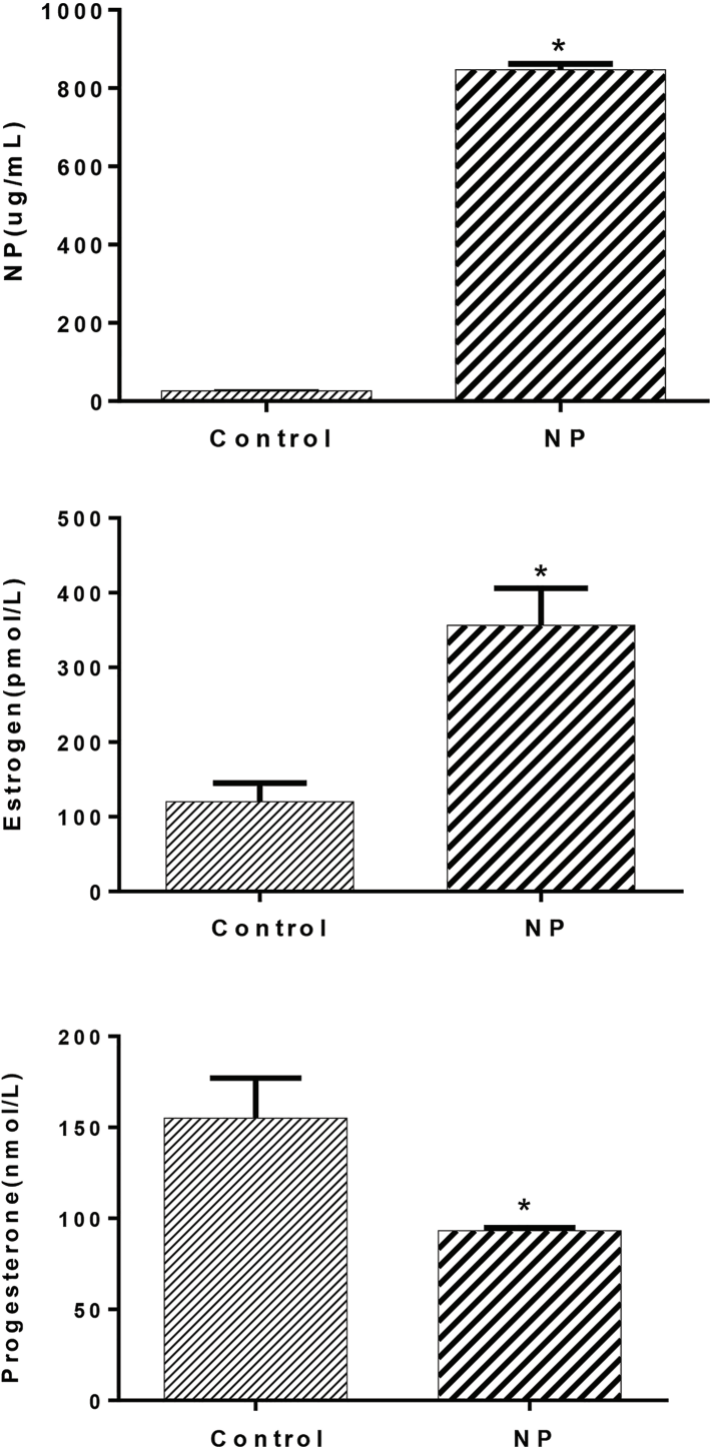
Serum parameters at 12 days of pregnancy in rats administered nonylphenol (NP) or corn oil (control) by gavage from the sixth day of pregnancy. Data are reported as means±SD. *P<0.05 *vs* control (*t*-test).

**Figure 3:**
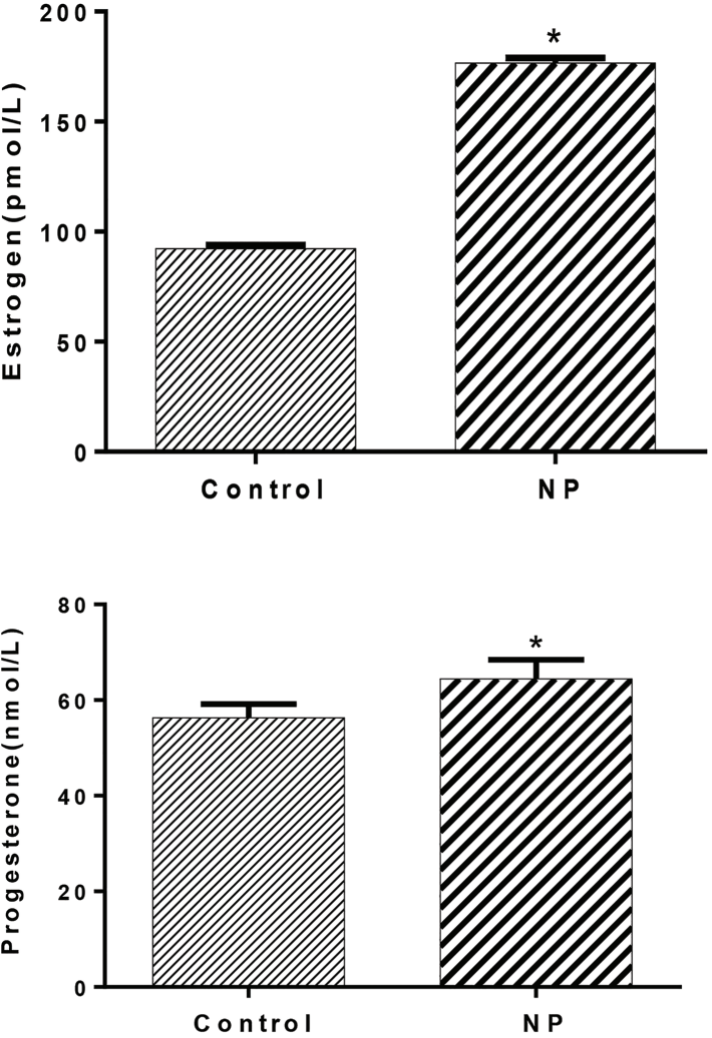
Serum hormone levels 1 day after giving birth of pregnant rats administered nonylphenol (NP) by gavage from the sixth day of pregnancy to 21 days postpartum or corn oil (control). Data are reported as means ±SD. *P<0.05 *vs* control (*t*-test).

Compared with the control group, the levels of NP and estradiol in the serum of the rats in the NP group at 12 days of pregnancy and 1 day after delivery were significantly higher (P<0.05) (Figure 2A and B). The progesterone level in the NP group was lower than in the control group (P<0.05), and the level of progesterone decreased after giving birth (Figures 2C and 3B).

### Serum and hepatic levels of NP and hormones in newborn rats at 60 days of age

**Figure 4:**
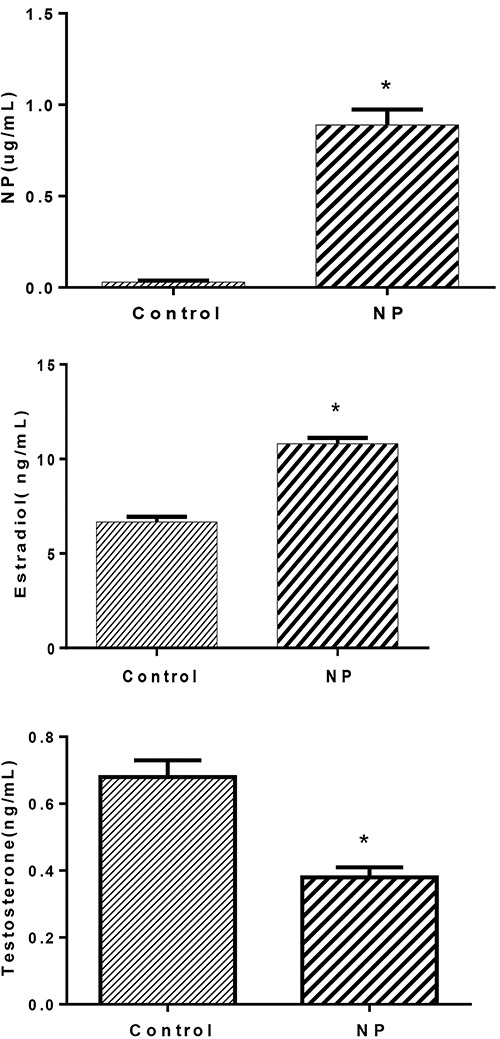
Liver levels of nonylphenol (NP) and hormones in newborn rats at 60 days of age from pregnant rats administered NP or corn oil (control) by gavage from the sixth day of pregnancy to 21 days postpartum. Data are reported as means±SD. *P<0.05 *vs* control (*t*-test).

The levels of NP in serum and liver of the newborn rats in the NP group were significantly higher than in the control group (P<0.05). Compared with the control group, the levels of testosterone were significantly decreased, while estradiol levels were significantly increased (Figure 4).

### Liver function and blood lipids of the newborn rats

**Figure 5:**
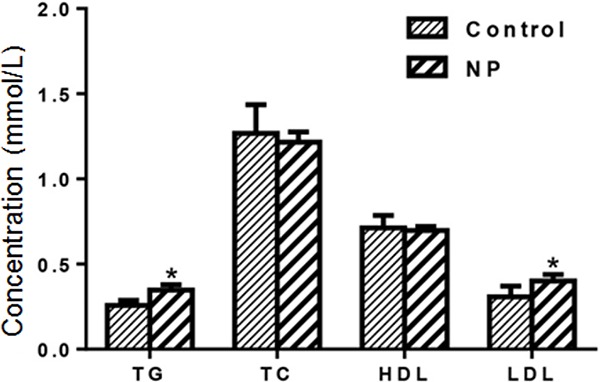
Blood lipid homeostasis in pregnant rats administered nonylphenol (NP) or corn oil (control) by gavage from the sixth day of pregnancy to 21 days postpartum. Data are reported as means ±SD. *P<0.05 *vs* control (*t*-test). TG: triglycerides; TC: total cholesterol; HDL: high-density lipoprotein; LDL: low-density lipoprotein.

**Figure 6:**
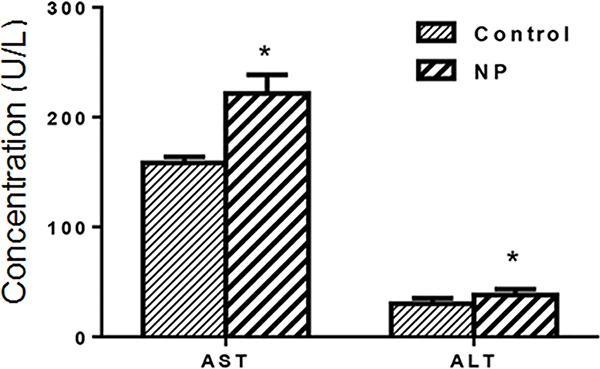
Liver function of newborn rats from pregnant rats administered nonylphenol (NP) by gavage from the sixth day of pregnancy to 21 days postpartum or corn oil (control). Data are reported as means ±SD. *P<0.05 *vs* control (*t*-test). ALT: alanine aminotransferase; AST: aspartate aminotransferase.

Compared with control group, the serum alanine aminotransferase (*t*=2.64, P<0.05), aspartate aminotransferase (*t*=8.59, P<0.05), triglyceride (*t*=5.19, P<0.05), and LDL (*t*=3.07, P<0.05) levels were higher in the NP group. There were no significant differences in cholesterol and HDL levels between the NP group and the control group (Figures 5 and 6).

### TNF-α and IL-1β in liver tissues

**Figure 7:**
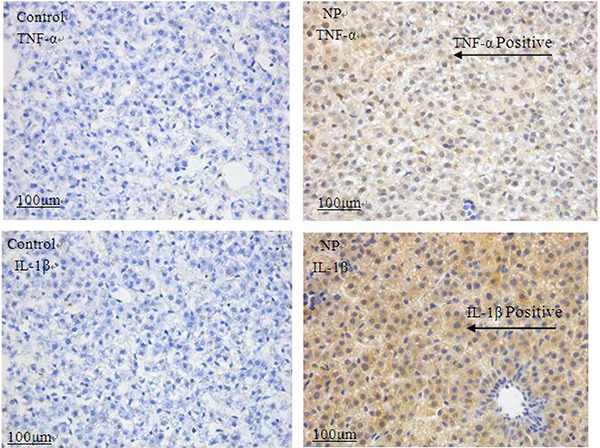
Expression of TNF-α (*top*) and IL-1β (*bottom*) in the liver of pregnant rats administered nonylphenol (NP) or corn oil (control) by gavage from the sixth day of pregnancy to 21 days postpartum, by immunohistochemistry.

**Figure 8:**
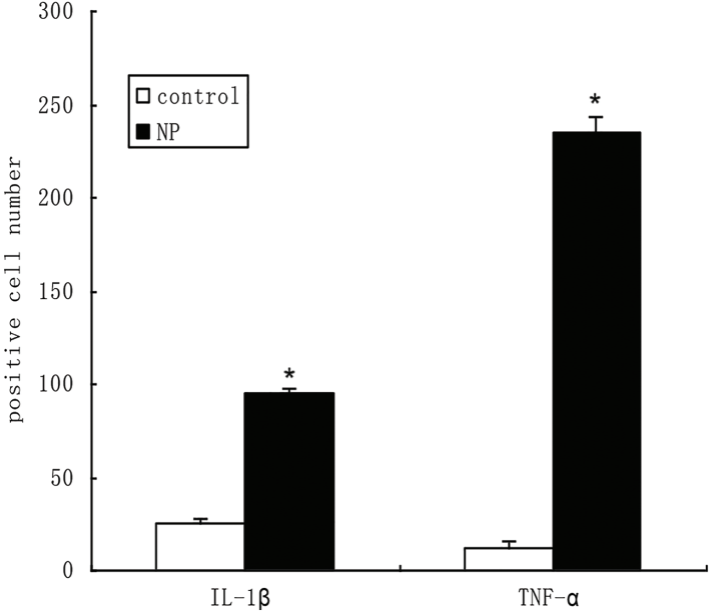
Number of TNF-α- and IL-1β-positive cells in liver tissue of pregnant rats administered nonylphenol (NP) or corn oil (control) by gavage from the sixth day of pregnancy to 21 days postpartum. Data are reported as means ±SD. *P<0.05 *vs* control (*t*-test).

**Figure 9:**
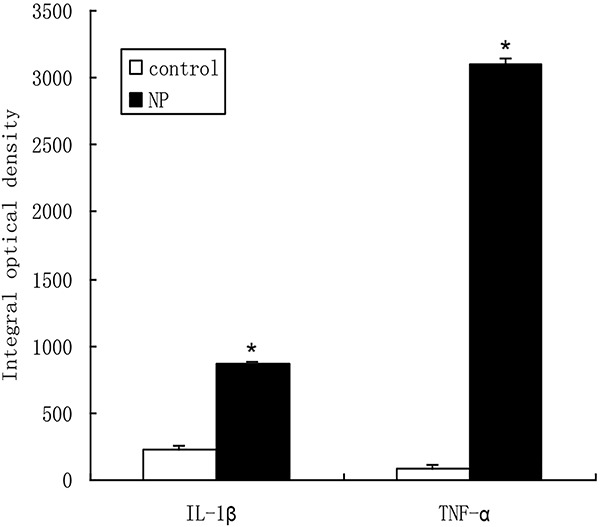
Integral optical density values of TNF-α and IL-1β in liver tissue of pregnant rats administered nonylphenol (NP) or corn oil (control) by gavage from the sixth day of pregnancy to 21 days postpartum. Data are reported as means±SD. *P<0.05 *vs* control (*t*-test).

There was almost no expression of TNF-α or IL-1β in the control group. The number of TNF-α and IL-1β positive cells was increased in the NP group, with positive staining sites in the cytoplasm (Figure 7A and B). Compared with the control group, the number of TNF-α and IL-1β positive cells increased, with higher IOD values (Figures 8 and 9).

### Ultrastructure of hepatocytes

**Figure 10:**
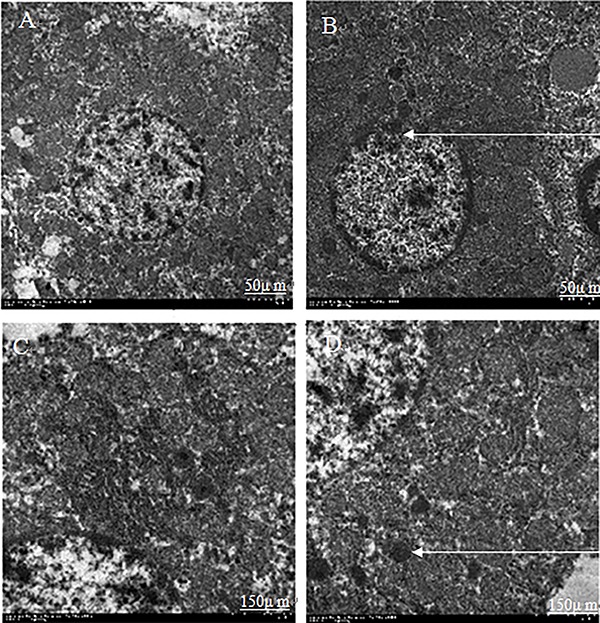
Hepatocytes of pregnant rats administered nonylphenol (NP) or corn oil (control) by gavage from the sixth day of pregnancy to 21 days postpartum in the control and NP groups under electron microscope. The arrows indicate lysosomal integral lipid droplets. *A*, Control group and *B*, NP group (×2000); *C*, control group and *D*, NP group (×2500).

Under the electron microscope, hepatocytes of the rats in the control group demonstrated large and round nuclei, with clear nuclear membrane and evenly distributed nuclear chromatin. In the NP group, nucleus chromatin in the hepatocytes was aggregated, with dark-stained nuclear membrane and a large number of lipid droplets in the cytoplasm, most of which were bond with lysate and deeply stained (Figure 10).

## Discussion

As a typical endocrine disruptor, through mimicking or interfering with synthesis, secretion, transport, binding and excretion of estrogen, NP can influence the body's physiological activities, demonstrating reproductive, carcinogenic, nervous, immune and metabolic toxicity (3,4). Studies have indicated that NP can pass through the placental barrier, and also enter breast milk. Therefore, compared with adults, embryos and infants in the development phase are more vulnerable to NP toxicity (5). To simulate the most common way of NP exposure, the current study administered NP by oral gavage. The pregnant rats were exposed to NP during the critical duration of fetal development, aiming to investigate the effects of perinatal NP exposure in the delivery of rats and the hepatotoxic effects on offspring.

Results showed that the food intake of pregnant rats in the NP group was reduced from the second day of NP administration (7 days of gestation), which is possibly due to the stomach discomfort of intragastric administration, or to pregnancy reaction; from the second week of pregnancy to prenatal, the body weight of the pregnant rats in both groups increased, with the NP group increasing less than the control group, indicating that NP has a certain toxicity to the pregnant rats. Ferguson et al. (6) reported that during pregnancy, animal food intake was reduced by 9∼15% after NP exposure, with body weight decreasing by 17%, which are consistent with the results of the current study. During gestation, most pregnant rats in the NP group suffered threatened abortion, vaginal bleeding or increased yellow secretion, which might be due to the hormone balance disorders caused by NP. Test results suggested that at 12 days of pregnancy, the estrogen level in the pregnant rats was too high while the progesterone level was too low. Estrogen can enhance uterine sensitivity to oxytocin, counteracting the stabilizing effect of progesterone to the uterus. In addition, serum NP content is positively correlated with estrogen levels, and negatively correlated with progesterone level, confirming that NP plays the role of estrogen by binding with various estrogen receptors, disrupting the body's normal endocrine function, leading to excessive estrogen secretion and to progesterone secretion deficiency, and subsequently causing threatened abortion symptoms in pregnant rats. Similarly, an epidemiological survey also showed that a high NP level in the blood and urine of 146 pregnant women at 27–38 gestational weeks played a certain role in pregnancy complications. On the other hand, the decreased breastfeeding after giving birth could be due to the breast underdevelopment due to insufficient progesterone secretion during the entire pregnancy. In this study, the female/male ratio of the newborn rats in the NP group was greater than the control group, which is consistent with our earlier study results (7 [Bibr B08]). The reason why NP exposure increased the proportion of female offspring needs to be further studied. In conclusion, perinatal exposure to NP increases NP levels in pregnant rats and leads to hormone balance disorders, affecting the delivery parameters of pregnant rats and postpartum breastfeeding, which is consistent with the conclusions of previous reports.

As for the NP-induced liver toxicity, studies have shown that 4-NP exposure increased hepatic lipid peroxidation levels, with reduced glutathione in catfish (15). The current study showed that triglyceride and LDL levels of the exposure group were increased. Electron microscopy observation showed increased fat accumulation in the hepatocytes of NP group, which is consistent with the study results of Maradonna et al. (9) on NP-induced liver injury and hepatic lipid accumulation. The mechanical study by Kim at al. further demonstrated that the adverse effects of NP on lipid metabolism of pregnant rats are associated with expression regulation of hepatic lipogenic gene *Fas* (fatty acid synthase) and *acc-1* (acetyl CoA carboxylase 1) (10). The current study verified such conclusion from the perspective of inflammatory factors. TNF- α and IL-1β are two major pro-inflammatory cytokines induced by oxidative stress and inflammation. The study by Dong et al. suggested that increased expression of TNF-α and IL-1β mRNA may be one of the steps in the pathogenesis of acute liver injury (11). Another research confirmed that TNF-α and IL-1β are important regulators of inflammatory and immune response, playing an important role in lipopolysaccharide-induced liver injury (12). However, NP-induced liver injury mediated by TNF-α and IL-1β has never been reported. Our results indicate, by measuring TNF-α and IL-1β levels, an inflammation reaction in the liver, suggesting that NP exposure during pregnancy and breastfeeding led to liver toxicity through increased liver pro-inflammatory cytokines. In conclusion, to protect children's health, it is very important to reduce NP intake during pregnancy and breastfeeding.
